# Immunization with Bivalent Flagellin Protects Mice against Fatal *Pseudomonas aeruginosa* Pneumonia

**DOI:** 10.1155/2017/5689709

**Published:** 2017-10-19

**Authors:** Bahador Behrouz, Farhad B. Hashemi, Mohammad Javad Fatemi, Sara Naghavi, Gholamreza Irajian, Raheleh Halabian, Abbas Ali Imani Fooladi

**Affiliations:** ^1^Applied Microbiology Research Center, Baqiyatallah University of Medical Sciences, Tehran, Iran; ^2^Department of Microbiology, School of Medicine, Tehran University of Medical Sciences, Tehran, Iran; ^3^Burn Research Center, Hazrat Fatima Hospital, Iran University of Medical Sciences, Tehran, Iran; ^4^Department of Bacteriology, Faculty of Medical Sciences, Tarbiat Modares University, Tehran, Iran; ^5^Department of Microbiology, School of Medicine, Iran University of Medical Sciences, Tehran, Iran

## Abstract

*Pseudomonas aeruginosa* lung infections present a major challenge to healthcare systems worldwide because they are commonly associated with high morbidity and mortality. Here, we demonstrate the protective efficacy of type a and b flagellins (bivalent flagellin) against acute fatal pneumonia in mice. Mice immunized intranasally with a bivalent flagellin vaccine were challenged by different flagellated strains of *P. aeruginosa* in an acute pneumonia model. Besides the protective effect of the vaccine, we further measured the host innate and cellular immunity responses. The immunized mice in our study were protected against both strains. Remarkably, active immunization with type a or b flagellin significantly improved survival of mice against heterologous strain compared to flagellin a or b antisera. We also showed that after an intranasal challenge by *P. aeruginosa* strain, neutrophils are recruited to the airways of vaccinated mice, and that the bivalent flagellin vaccine was proved to be protective by the generated CD4^+^IL-17^+^ Th17 cells. In conclusion, bivalent flagellin vaccine can confer protection against different strains of *P. aeruginosa* in an acute pneumonia mouse model by eliciting effective cellular and humoral immune responses, including increased IL-17 production and improved opsonophagocytic killing.

## 1. Introduction

*Pseudomonas aeruginosa* pulmonary infection as a life-threatening complication frequently causes bacteremia and sepsis in hospitalized and immunocompromised patients [1, 2]. Moreover, the widespread and empirical use of broad-spectrum antibiotics in critical care units has led to the continuous emergence of multidrug-resistant (MDR) *P. aeruginosa* strains that present a major challenge to clinical therapy and contribute significantly to increased morbidity and mortality [3]. The high mortality and prevalence of infection with MDR *P. aeruginosa* strains accompanied by the paucity of new effective antibiotic classes present unique challenges to clinicians and highlight the necessity for designing new treatment approaches, such as immunotherapy, which targets pathogen-specific virulence factors to reduce pathogenesis without inducing multidrug resistance [4, 5]. Moreover, the complexity of the *P. aeruginosa* genome, which encodes numerous antigens, indicates that immunotherapy using a single antigen will not provide sufficient protection.

To date, most *P. aeruginosa* vaccines focusing on immunization and treatment targeting pseudomonal virulence factors, such as LPS O antigen [6], the outer membrane proteins F and I [7], or the type III secretion system component PcrV [8], have been described in the literature as having conventional protective mechanisms, namely, antibody-mediated opsonophagocytic killing and/or antibody-mediated toxin inhibition. Recently, studies have shown that Th17 cells are critical for providing protection against *P. aeruginosa* pneumonia via rapid recruitment of neutrophils to the airways in the absence of opsonophagocytic antibody [9, 10]. These findings suggest that an effective vaccine for providing full-fledged protection against various *P. aeruginosa* infections in different tissues can induce multiple cellular and humoral effectors.

Most clinical isolates of *P. aeruginosa* are motile with the aid of a single polar flagellum, which is essential for systemic spread throughout the organs from the initial site of colonization [11]. Flagellin is the primary protein component of the flagella, which is classified into two distinct serotypes a and b [12]. Furthermore, the conserved domains of flagellin are strongly antigenic and serve as a pathogen-associated molecular pattern (PAMP) that induce a strong NF*κ*B-mediated inflammatory response via toll-like receptor 5 (TLR5) signal transduction [13]. Several studies have shown the strong immunogenicity of flagellin initiates a general recruitment of both T and B cells to secondary lymphoid sites, eliciting TLR5^+^, CD11c^+^, and T cell-like similar antigen recognition [14, 15]. Due to TLR5 agonist activity of flagellin, they play an important role as potent adjuvants to enhance protective humoral responses [15, 16]. Several *in vivo* studies not only have demonstrated the importance of flagellins as a crucial virulence factor contributing to the pathogenesis of lung infections but also validated them as target antigens for immunization [17–22]. Immunization with type a or b flagellin provided protection against lung infections [23], keratitis [24], urinary tract, and burn wound infections [25]. These studies have confirmed that protection provided by flagellin is highly type specific and the presence of both types of flagellin is crucial. The results of our recent studies [20, 26] demonstrating protection provided by flagellin in *P. aeruginosa* burn infection via induction of IL-17 encouraged us to study active and passive immunization strategies using bivalent flagellin in order to provide full-fledged protection against various *P. aeruginosa* clinical isolates in the acute fatal pneumonia model.

## 2. Materials and Methods

### 2.1. Bacterial Strains

*P. aeruginosa* strains PAK and PAO1 were used for the purification of type a and b flagellin proteins, respectively.

### 2.2. Animals

Female 6–8-week-old BALB/C mice were purchased from Pasteur Institute (Tehran, Iran). All animal experiments were complied with institutional animal care committee (IACC) guidelines regarding the use of animals in research.

### 2.3. Preparation of Recombinant Proteins

Recombinant type a and b flagellin proteins were purified from *E. coli* BL21 (DE3) carrying pET-28a vector as previously described [27, 28].

### 2.4. Immunization of Mice

Mice were immunized intranasally (i.n.) as described previously. Briefly, mice were anesthetized by mixture of ketamine (6.7 mg/ml) and xylazine (1.3 mg/ml) and then immunized by placing 10 *μ*l of mixed flagellins (1 *μ*g of each flagellin), or flagellin a (2 *μ*g), or flagellin b (2 *μ*g), or PBS on each nostril at weekly intervals. In several published *P. aeruginosa* vaccine studies, PBS has been used as a control [9, 29–31]. At day 42, mice were challenged i.n. with 2 × 10^7^ CFU of *P. aeruginosa* strains directly into each nostril, as described previously [23]. In addition, five mice from each group were sacrificed 24 h after infection. Then the lung, liver, blood, and spleen were harvested for bacterial load enumeration. Bronchoalveolar lavage fluid (BALF) was collected 6 and 18 h after infection for quantitation of CFU, neutrophils, and IL-17 cytokine. For passive immunization, mice were injected intraperitonealy (i.p.) with 200 *μ*l of antibodies raised to bivalent flagellin, or flagellin a, or flagellin b 12 h before and after challenge. All mice were closely monitored for one week.

### 2.5. Enzyme-Linked Immunosorbent Assay (ELISA)

To determine the levels of specific antibodies (total IgG, IgG1, and IgG2a) in mouse sera, ELISA was performed with plates coated with a whole live cell of *P. aeruginosa* strains PAK or PAO1 or mixed flagellins as described previously [20]. Briefly, 100 *μ*l of either *P. aeruginosa* strains PAK or PAO1 or mixed flagellins was incubated overnight at 4°C, washed with 0.5% Tween-PBS (T-PBS), and blocked with PBS + 3% bovine serum albumin (Sigma-Aldrich). Indicated dilutions of sera from immunized mice were incubated overnight at 4°C and washed 3x with T-PBS, and 100 *μ*l of 1 : 7000-diluted horseradish peroxidase-conjugated goat anti-mouse (HRP; Sigma-Aldrich) was incubated (1 h, RT). Plates were then washed five times with T-PBS, and of TMB substrate (Sigma-Aldrich) was added (100 *μ*l/well; 30 min. at RT). Concentrations of serum IgG1 and IgG2a subtypes against mixed flagellins were determined as described above, except that we used 100 *μ*l/well of IgG subtype-specific HRP-conjugated secondary anti-mouse IgG1, or IgG2a antibody (Sigma-Aldrich) diluted 1 : 8000 in 0.5% skim milk/T-PBS.

### 2.6. Cell Proliferation and Cytokine Measurements

For lymphocyte proliferation assays, ELISA plates were seeded with 1 × 10^5^ T cells, 1 × 10^5^ irradiated (1500 rad) splenocytes isolated from naïve mice as antigen-presenting cells (APCs), and 1 *μ*g of flagellin a, or flagellin b as antigen, while other groups contained 1 *μ*g per well anti-CD4, anti-CD8, and rat IgG isotype (all are from BD Biosciences) antibody. The cells were all cultured in RPMI 1640 containing 10% heat-inactivated FBS. At 24 h and 72 h after incubation, T lymphocyte proliferation assay was carried out using 5-bromo-2-deoxyuridine (BrdU; Roche, Germany) according to the manufacturer's protocol. At the same time, IL-17 levels were measured by examining cell-free culture supernatant fluid using specific ELISA assay (R&D Systems).

### 2.7. Depletion of Immune Effectors

Immune cell subsets were depleted as described [32]. For *in vivo* CD4 or CD8 T cells, T cell depletion, CD4-specific mAb (GK1.5; BD PharMingen), or CD8-specific mAb (53-6.7; BD PharMingen) were administered both intranasally and intraperitoneally (100 *μ*g/dose and 500 *μ*g/dose, resp.) 72 and 24 hours before bacterial challenge. Depletion of the targeted cell types was confirmed by subsequent FACS analysis, which showed >98% depletion of the respective cell type. Polyclonal antibody to murine IL-17 was produced by immunizing rabbits at multiple intradermal sites with mouse recombinant IL-17 (R&D Systems) mixed with CFA, as previously described [33]. Affinity chromatography (using protein G) was used for the separation of anti-IL-17 IgG from the whole serum, according to the manufacturers' instructions (Thermo Fisher Scientific, USA). This antibody was specific for IL-17, as determined by ELISA, but did not cross-react IL-2, IFN-*γ*, IL-4, IL-10, IL-5, or IL-13. The control antibody used in these experiments was the IgG fraction from normal rabbit serum purified by protein G affinity chromatography, as described above. IL-17 depletion studies were done using anti-IL-17 IgG (1 mg i.p.) or control IgG for 3 consecutive days before the mice were challenged by *P. aeruginosa* strains as described by Li et al. [34].

### 2.8. Opsonophagocytic Activity Assay

The assay was performed as described [20, 25]. Briefly, assays were performed in a sterile microcentrifuge tube 100 *μ*l of each component, polymorphonuclear leukocytes (PMNs; 2 × 10^9^ cells), *P. aeruginosa* strains (5 × 10^7^ CFUs), infant rabbit serum, and diluted antibodies. Generally, ≥50% opsonic killing activity of immune serum is considered biologically significant [10].

### 2.9. Motility Inhibition Assay

Assays were performed as described [20, 25]. Briefly, antibodies were added to motility agar (LB with 0.3% (*w/v*) agar) in 24-well plates (Greiner Bio-One, Germany). *P. aeruginosa* strains (OD_600_ = 0.2) were added into the central well of each plate and incubated at 37°C. Mean diameters of bacterial colonies with sharp and less distorted rings were measured after incubating for 18 h.

### 2.10. Statistical Analysis

All statistical analyses were performed using GraphPad Prism 6 (GraphPad Software Inc., USA). Nonparametric data were analyzed by Kruskal-Wallis followed by Dunn's multiple comparison tests. Parametric data were evaluated by ANOVA with Tukey's multiple comparison tests. All results were expressed as the mean ± standard deviation (SD). The log-rank test was used to compare Kaplan-Meier between different treatment mouse groups. *P* value of <0.05 was considered statistically significant.

## 3. Result

### 3.1. Protective Efficacy of Bivalent Flagellin Immunization

**Figure 1:**
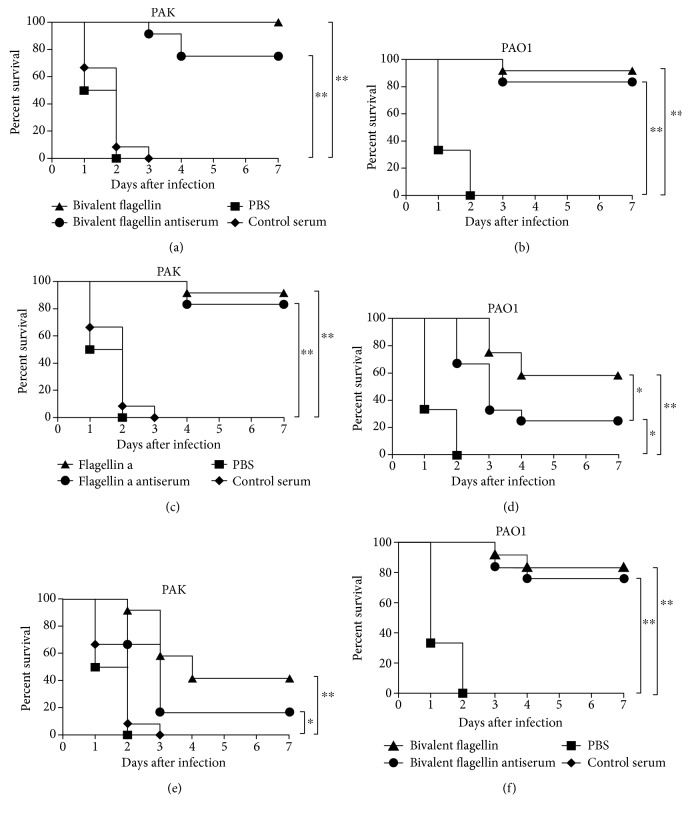
Survival rates of actively and passively immunized BALB/c mice (*n* = 12 mice/group) to infection with *P. aeruginosa* strains. Active and passive immunization against bivalent flagellin (a, b), flagellin a (c, d), and flagellin b (e, f). Kaplan-Meier curves were plotted for mice of the above groups, which were challenged by 2 × 10^7^ CFUs of *P. aeruginosa* strains PAO1 and PAK and monitored the seven day survival rates. ^∗^*P* < 0.05 and ^∗∗^*P* < 0.01.

To determine the protective efficacy of the bivalent flagellin vaccine against *P. aeruginosa* strains, immunized mice were challenged i.n. with different flagellated strains of *P. aeruginosa*. As shown in Figures 1(a) and 1(b), nasal immunization with bivalent flagellin protected against lethal pneumonia due to PAK (100% survival) and PAO1 (91.66% survival) and survival rates were all significantly higher than those in mice administered with PBS (*P* > 0.01). Flagellin a-immunized mice were significantly protected (*P* > 0.05) from lethality after challenge with homologous strain PAK with a survival rate of 91.66%, compared to 58.33% for heterologous strain PAO1 (Figures 1(c) and 1(d)). Nasal immunization with flagellin b leads to 83.33% protective effect for homologous strain PAO1 compared with 41.66% in the heterologous PAK challenge (*P* > 0.05; Figures 1(e) and 1(f)). However, provided heterologous protection by flagellin a or flagellin b immunization was significantly higher than that of the controls.

**Figure 2:**
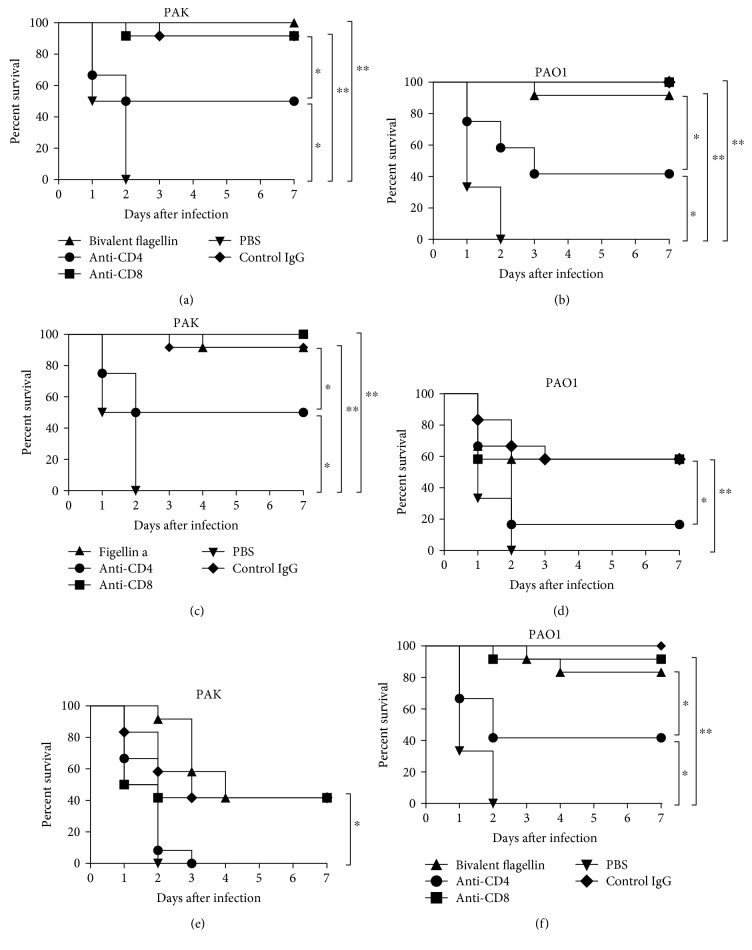
The protective role of CD4^+^ T lymphocytes in bivalent flagellin vaccine against *P. aeruginosa* strains. Bivalent flagellin- (a, b), flagellin a- (c, d), and flagellin b- (e, f) immunized mice (*n* = 12 mice/group) were treated with either anti-CD8 monoclonal antibody, or anti-CD4 monoclonal antibody, or normal rat IgG and then were challenged with 2 × 10^7^ CFUs of *P. aeruginosa* strains PAO1 and PAK. ^∗^*P* < 0.05 and ^∗∗^*P* < 0.01. NS = nonsignificance.

Moreover, we evaluated the efficacy of passive immunization with antisera from bivalent flagellin-, or flagellin a-, or flagellin b-, or PBS-immunized mice against *P. aeruginosa* strains. Bivalent flagellin antisera completely protected mice from challenge with PAK (75% survival) and PAO1 (83.33% survival; Figures 1(a) and 1(b)). Transfer of flagellin a antisera exhibited significant protective effect role against homologous strain PAK (83.33% survival, *P* < 0.01) but only has a partial protective role against heterologous strain PAO1 (25% survival, Figures 1(c) and 1(d)). Passive immunization with flagellin b significantly protected immunized mice against homologous strain PAO1 (75% survival, *P* < 0.01) compared with heterologous strain PAK (16.66% survival, Figures 2(e) and 2(f)). However, provided heterologous protection by antisera to flagellin a or flagellin b was significantly higher than that of the controls (*P* < 0.05; Figures 1(c), 1(d), 1(e), and 1(f)).

### 3.2. Opsonic Killing Activity of Bivalent Flagellin Immune Sera

**Figure 3:**
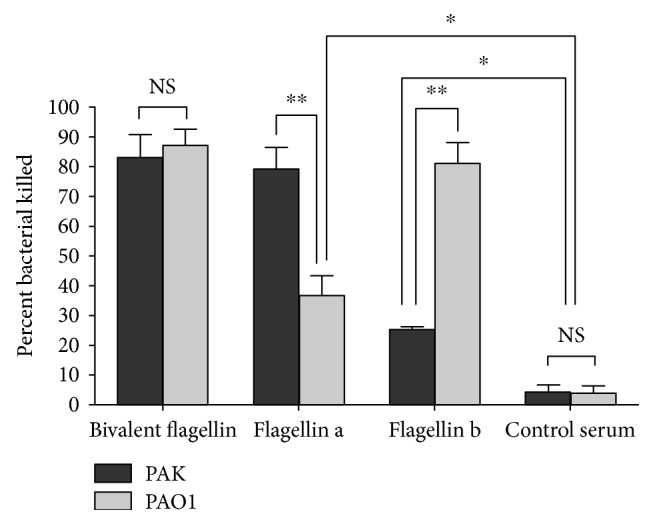
The opsonic killing activity of sera from bivalent flagellin-, flagellin a-, and flagellin b-immunized mice 3 weeks after the final immunization. Values presented as the mean of triplicate technical replicates ± SD. ^∗^*P* < 0.05 and ^∗∗^*P* < 0.01. NS = nonsignificance.

To test the functional activity of serum antibodies in the protection afforded by the bivalent flagellin vaccine, we evaluated the *in vitro* opsonic killing activity of pooled sera obtained from immunized mice 3 weeks after the third (final) nasal immunization. As shown in Figure 3, bivalent flagellin antisera at a dilution 1 : 10 significantly promoted the phagocytosis of *P. aeruginosa* strains PAK (82.70%) and PAO1 (86.23%) compared with that of serum isolated from control group mice (*P* < 0.01). Flagellin a antiserum in the immune serum showed significantly higher killing ability against homologous strain PAK (78.61%, *P* < 0.01) in comparison with PAO1 strain (33.56%, Figure 3). Sera from flagellin b-immunized mice had significantly killing activity against homologous strain PAO1 (80.59%, *P* < 0.01), comparable to that against heterologous strain PAK (24.27%, Figure 3). However, the opsonic killing activity of flagellin a or flagellin b antisera against the heterologous strain was significantly higher than that of serum from control mice (*P* < 0.05).

### 3.3. Antimotility Activity of Bivalent Flagellin Immune Sera

**Figure 4:**
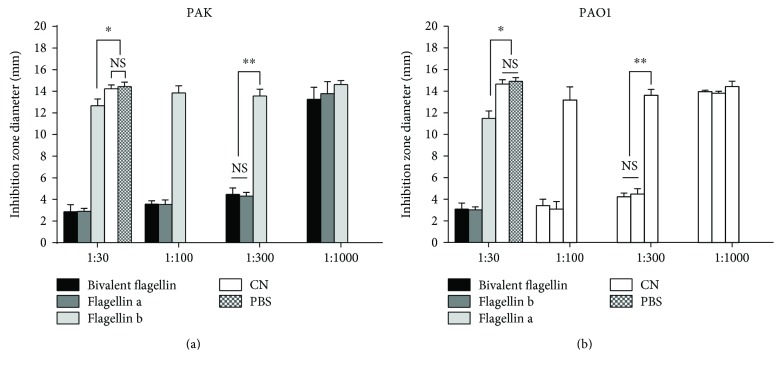
Assessment of motility inhibition of *P. aeruginosa* strains PAK (a) and PAO1 (b) by different dilutions of sera from bivalent flagellin-, flagellin a-, or flagellin b-immunized mice. Motility agar plates are prepared with diluted antibodies in each well prior to stabbing each well with fresh overnight bacterial cultures. The mean diameters of *P. aeruginosa* strains were measured in millimeters (mean + SD). Values presented as mean of triplicate independent experiments ± SD. ^∗^*P* < 0.05 and ^∗∗^*P* < 0.01. NS = not significant. CS = control serum.

We evaluated the functional activity of bivalent flagellin immune sera to inhibit the motility of different flagellated *P. aeruginosa* strains. As shown in Figures 4(a) and 4(b), bivalent flagellin immune sera at a dilution of 1 : 300 or less completely inhibited the motility of *P. aeruginosa* strains compared with the controls (*P* < 0.01). Antisera to flagellin a at dilution of 1 : 300 or less completely inhibited the motility of the homologous strain (*P* < 0.01), but there was some cross-reactivity with the heterologous strain (*P* < 0.05; Figures 4(a) and 4(b)). Antibodies against flagellin b at dilution of 1 : 300 or less inhibited the motility of homologous strain PAO1 more significantly than heterologous strain PAK (*P* < 0.05; Figures 4(a) and 4(b)).

### 3.4. Specificity of Antibodies Raised against Bivalent Flagellin

**Figure 5:**
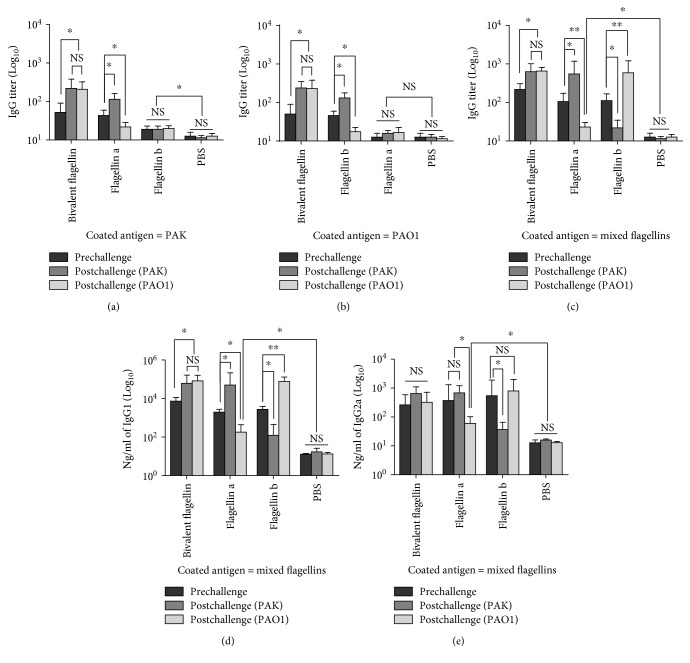
Effects of bivalent flagellin immunization on pre and postchallenge levels of serum total IgG against the whole cell of *P. aeruginosa* PAK (a), PAO1 (b), and mixed flagellins (c). The pre- and postchallenge serum IgG1 (d) and IgG2a (e) titers against mixed flagellins. Values are represented as mean ± SD based on five mice in each group. ^∗^*P* < 0.05 and ^∗∗^*P* < 0.01. NS = nonsignificance.

To assess the humoral responses evoked by bivalent flagellin vaccine, we compared serum IgG levels among immunized mice challenged with either the PAK or PAO1 strain. While the prechallenge IgG levels of mice injected with bivalent flagellin against whole live cells of *P. aeruginosa* strains or mixed flagellins were similar, the serum IgG levels significantly increased after infection with PAK or PAO1 strains infection (*P* < 0.05; Figures 5(a), 5(b), and 5(c)). IgG titer in sera from vaccinated mice with type a or b flagellin significantly increased after challenge with the homologous strain compared with that in heterologous strain of *P. aeruginosa* infection (*P* < 0.01; Figures 5(a), 5(b), and 5(c)). Similar results were obtained when IgG1 subtype was examined (Figure 5(d)). Among bivalent flagellin-immunized mice, no significant differences in the titer increases for specific IgG2a subtypes against mixed flagellins were observed in pre- and postchallenge with PAK or PAO1, respectively (*P* > 0.05; Figure 5(e)). IgG2a level in mice immunized with type a or b flagellin was significantly decreased after challenge with the heterologous strain compared with mice challenged with the heterologous strain (*P* < 0.05; Figure 5(e)). The titer of antigen-specific antibodies induced by bivalent flagellin was similar to that induced by flagellin a or b, which indicated that there is no interference among each vaccine component in induction of protective antibodies (data not shown).

### 3.5. T Cell Responses Induced by Bivalent Flagellin Vaccine

**Figure 6:**
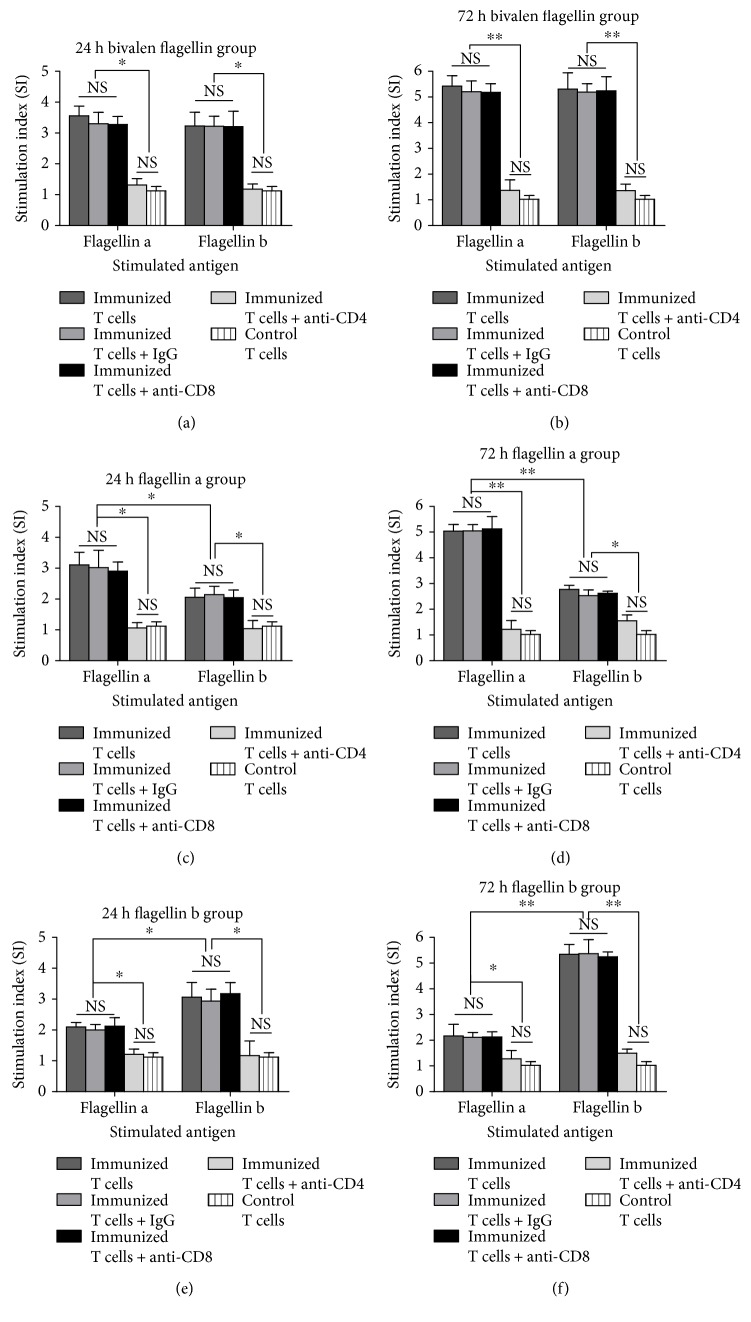
Proliferative assay of immune splenic T cells from bivalent flagellin (a, b), flagellin a (c, d), and flagellin b (e, f) stimulated with antigens *in vitro* for 24 h and 72 h. Values are represented as mean ± SD based on five mice in each group. ^∗^*P* < 0.05 and ^∗∗^*P* < 0.01. NS = nonsignificance.

To test whether vaccine candidates could induce lymphocyte proliferation, we analyzed the stimulation index of splenic T cells from immunized mice. As shown in Figures 6(a) and 6(b), there was a higher proliferation level of bivalent flagellin immune T cells to either flagellin a or flagellin b at both 24 and 72 h when compared with T lymphocytes isolated from the controls (*P* < 0.01), which means vaccinated T lymphocytes could be stimulated by both types of flagellin without interference. (Figures 6(a) and 6(b); *P* < 0.05). The proliferation rate of flagellin a or b immune T cell cultures stimulated with the homologous antigen at both 24 and 72 h was significantly higher than the cultures stimulated with heterologous antigen (Figures 6(c) and 6(d) and Figures 6(e) and 6(f); *P* < 0.01). Additionally, the proliferation level of flagellin a or b immune T cell cultures stimulated with heterologous antigen was significantly higher than T cells from control mice (Figures 6(c) and 6(d) and Figures 6(e) and 6(f); *P* < 0.05).

In order to further distinguish whether CD4^+^ T lymphocytes were the immune cells involved in the anti-infection effect by bivalent flagellin vaccine *in vivo*, CD4^+^ or CD8^+^ T lymphocytes were depleted independently by corresponding antibodies as described above. We demonstrated that anti-infection activity with the bivalent flagellin immunization relatively abrogate in *in vivo* depletion of CD4^+^ T lymphocytes. After depletion of CD4^+^ T lymphocytes in the bivalent flagellin-immunized group, the survival rate decreased to 50% and 41.66% when infected with strain PAK and PAO1, respectively (Figures 2(a) and 2(b)). Also, the survival rate was not decreased compared to the control group when challenged by PAK (Figure 2(a)) and PAO1 (Figure 2(b)), since the killing ability of immune serum against *P. aeruginosa* strains was effective. Depletion of CD4^+^ T lymphocytes in flagellin a-immunized group decreased the survival rate to 50% and 16.16% when challenged with strain PAK and PAO1, respectively (Figures 2(c) and 2(d)). The survival rate of mice in flagellin b-immunized group after depletion of CD4^+^ T lymphocytes decreased to 41.66% and 0% when challenged with the strain PAO1 and PAK, respectively (Figures 2(e) and 2(f)). Depletion of CD8^+^ T lymphocytes showed no effect on the survival rate of immunized mice. Also, the treatment with normal rat IgG showed no effect on the survival rate.

### 3.6. Th17 Cells and IL-17 Were Activated by Bivalent Flagellin Vaccine

**Figure 7:**
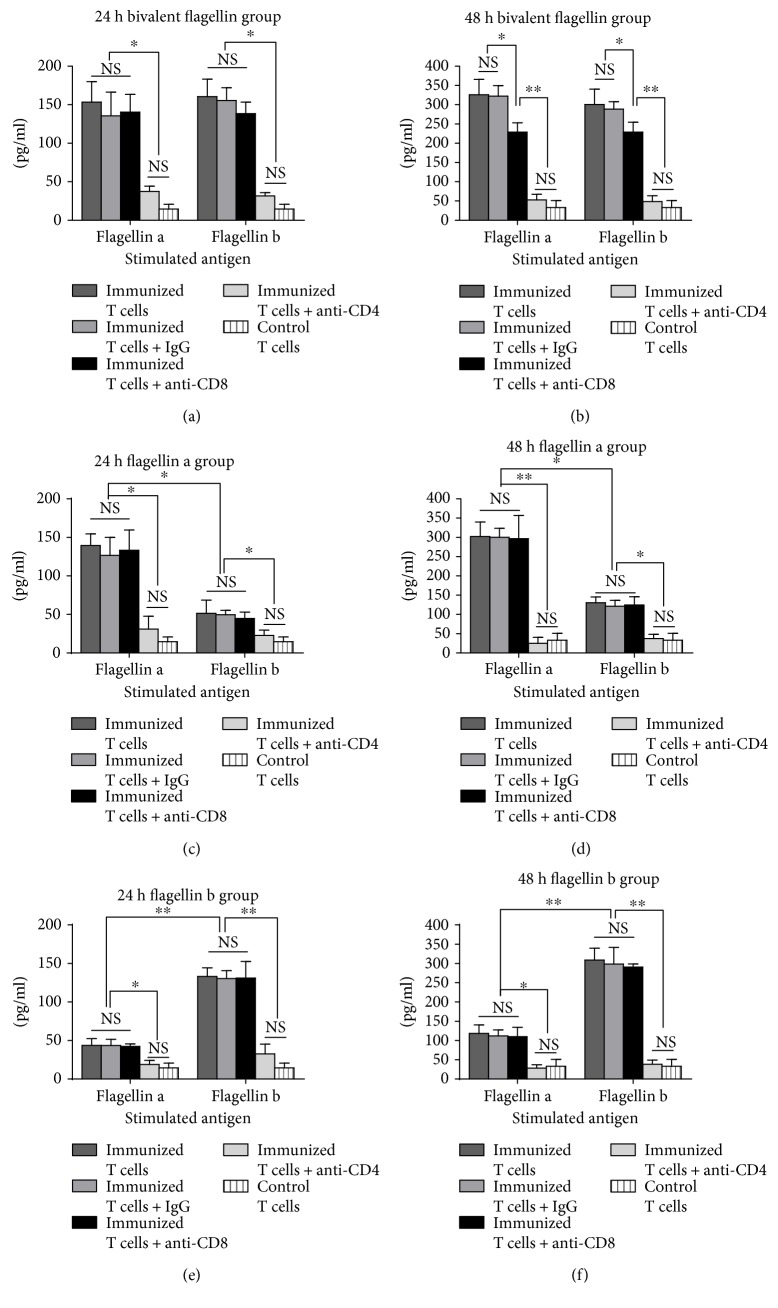
Effects of active immunization with bivalent flagellin on IL-17 production of immune splenic T cells from bivalent flagellin (a, b), flagellin a (c, d), and flagellin b (e, f) at 24 h and 48 h after stimulation with flagellin a or flagellin b. Values represented as mean ± SD based on five mice in each group. ^∗^*P* < 0.05 and ^∗∗^*P* < 0.01. NS = nonsignificance.

To test whether the bivalent flagellin vaccine could induce IL-17, we evaluated IL-17 production by the CD4^+^ T cells recovered from immunized mice in the presence of irradiated splenocytes and flagellin a or flagellin b. The levels of IL-17 in the supernatants of the immunized T cells were significantly higher than those of control T cells (*P* < 0.05, Figures 7(a) and 7(b)). The presence of the anti-CD4 monoclonal antibody during coculture returned the IL-17 levels as those of the control T cells, further indicating that CD4^+^ T cells are the predominant source of IL-17 in this system (Figures 7(a) and 7(b)). The IL-17 levels in the presence of the anti-CD8 monoclonal antibody during coculture had no significant difference between each group (Figures 7(a) and 7(b); *P* > 0.05). IL-17 production by flagellin a- or flagellin b-immunized T cells when stimulated with the homologous antigen was significantly higher than that of T cells stimulated with heterologous antigen (*P* < 0.05; Figures 7(c) and 7(d) and Figures 7(e) and 7(f)). IL-17 production by flagellin a- or flagellin b-immunized T cells when stimulated with heterologous antigen was significantly higher than that of the control T cells (*P* < 0.05; Figures 7(c) and 7(d) and Figures 7(e) and 7(f)). Flagellin a- or flagellin b-immunized T cells when stimulated with homologous or heterologous antigen in the presence of the anti-CD4 monoclonal antibody during coculture returned the IL-17 levels as those of the control T cells. There was no significant difference between the IL-17 levels of flagellin a or flagellin b among cultures treated by either immunized T cells, anti-CD8 monoclonal antibody, or control IgG (*P* > 0.05; Figures 7(c) and 7(d) and Figures 7(e) and 7(f)). T cell proliferation and IL-17 secretion response assays were confirmatory data showing the evidence for a heterologous T cell response among immunized flagellin a or b mice indicating an active clonal expansion and a neutrophil response that lead to higher survival (protective) rates among immunized mice than control mice.

### 3.7. Bivalent Flagellin Vaccine Decreased *P. aeruginosa* Strain Burden

**Figure 8:**
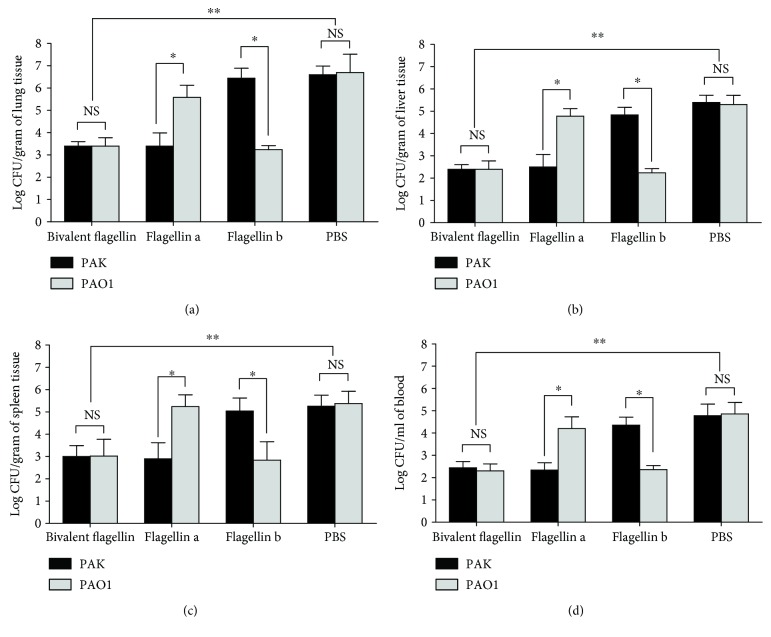
The effect of nasal immunization with bivalent flagellin, flagellin a, and flagellin b on the local and systemic spread of *P. aeruginosa* strains PAK and PAO1. Bacterial load was determined as CFUs (log) in the lung (a), liver (b), spleen (c), and blood (d). Values represented as mean ± SD based on five mice in each group. ^∗^*P* < 0.05 and ^∗∗^*P* < 0.01. NS = nonsignificance.

In order to evaluate the protective efficacy of bivalent flagellin vaccine, we determined the bacteria number in the lung, liver, blood, and spleen of infected immunized mice. Immunization with bivalent flagellin reduced the PAK and PAO1 strain burden in the lung, liver, blood, and spleen compared with mice injected with PBS (*P* < 0.01; Figures 8(a), 8(b), 8(c), and 8(d)). Immunization with flagellin a significantly decreased homologous strain PAK burden in the main organs and blood compared with the heterologous strain PAO1 burden (*P* < 0.05; Figures 8(a), 8(b), 8(c), and 8(d)). Immunization with flagellin a significantly decreased homologous strain PAO1 burden in the main organs and blood compared with heterologous strain PAK burden (*P* < 0.05; Figures 8(a), 8(b), 8(c), and 8(d)).

### 3.8. Neutrophil Recruitment to the Lungs of Bivalent Flagellin*-*Immunized Mice

**Figure 9:**
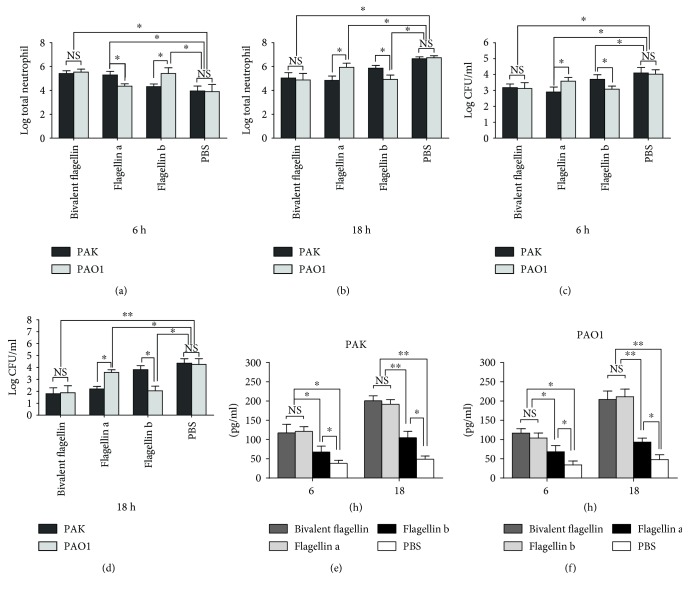
Total BALF neutrophils at 6 h (a) and 18 h (b) after infection of immune and nonimmune mice with *P. aeruginosa* strains PAK and PAO1. The bacterial CFU in BALF at 6 h (a) and 18 h (b) after infection of immune and nonimmune mice with PAK (e) and PAO1 (f). IL-17 level in BALF at 6 h (a) and 18 h (b) after infection of immune and nonimmune mice with PAK (e) and PAO1 (f). Values represented as mean ± SD based on five mice in each group. ^∗^*P* < 0.05 and ^∗∗^*P* < 0.01. NS = nonsignificance.

As IL-17 is a critical mediator for neutrophil recruitment to the lung, we evaluated the numbers of neutrophils recruited to the airways in immunized mice. As shown in Figures 9(a), 9(e), and 9(f), in bivalent flagellin-immunized mice, the number of BALF neutrophils and IL-17 levels at 6 h after the challenge were significantly more than those of the controls (*P* < 0.05). At this point in time, the bacteria numbers in the BALF of bivalent flagellin-immunized were significantly lower than those of the controls (*P* < 0.05, Figure 9(c)). IL-17 levels and neutrophil numbers in BALF of the flagellin a- or b-immunized mice obtained 6 h after homologous challenge were significantly higher than those in mice challenged with the heterologous strain (*P* < 0.05; Figures 9(a), 9(e), and 9(f)). In flagellin a- or b-immunized mice, IL-17 levels and neutrophil numbers in BALF obtained 6 h after challenge with the heterologous strain were significantly higher than those in nonimmunized mice (*P* < 0.05; Figures 9(a), 9(e), and 9(f)). At this point in time, the heterologous strain numbers in the BALF of flagellin a- or b-immunized mice were significantly lower than those in mice challenged with the heterologous strain. (*P* < 0.05, Figure 9(c)). At 18 h after bacterial challenge, the number of BALF neutrophils and bacterial CFU in bivalent flagellin-immunized mice was significantly lower than that of the controls (*P* < 0.05; Figures 9(b) and 9(d)). IL-17 levels and neutrophil in BALF of the flagellin a- or b-immunized mice obtained 18 h after homologous challenge were significantly higher than those in mice challenged with the heterologous strain (*P* < 0.05; Figures 9(b), 9(e), and 9(f)). At this point in time, the heterologous strain numbers in the BALF of flagellin a- or b-immunized mice were significantly lower than those in mice challenged with the heterologous strain. (*P* < 0.05, Figure 9(d)).

### 3.9. Effect of Neutralization of IL-17 on Protective Efficacy of Bivalent Flagellin Vaccine

**Figure 10:**
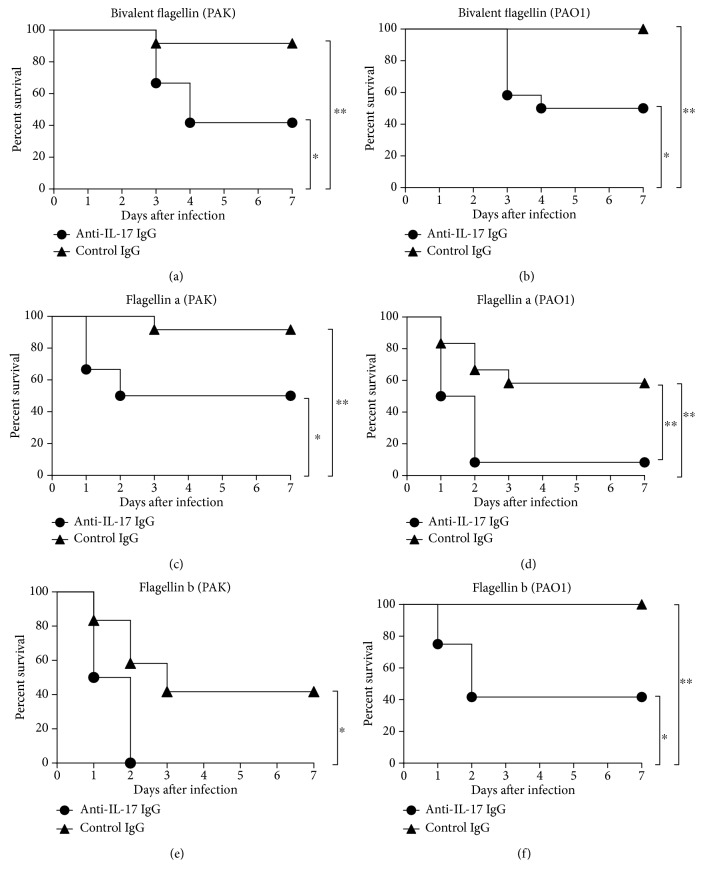
The role of IL-17 in vaccine based protection against *P. aeruginosa* strains. Bivalent flagellin- (a, b), flagellin a- (c, d), and flagellin b- (e, f) immunized mice (*n* = 12 mice/group) were given IL-17 IgG or control IgG for 3 consecutive days prior to challenge with 2 × 10^7^ CFUs of *P. aeruginosa* strains PAO1 and PAK. ^∗^*P* < 0.05 and ^∗∗^*P* < 0.01.

To further assess the protective role of IL-17 in the bivalent flagellin vaccine efficacy, we investigated the effects of neutralization of IL-17 prior to lung challenge with *P. aeruginosa* strains. As shown in Figures 10(a) and 10(b), mortality in the bivalent flagellin-immunized mice which received anti-IL-17 IgG was significantly higher than that given in the control IgG (*P* < 0.05). In flagellin a-immunized group, the survival rate decreased to 50% and 8.33% after receiving anti-IL-17 IgG when challenged with the strains PAK and PAO1, respectively (Figures 10(c) and 10(d)). In flagellin b-immunized group, the survival rate decreased to 41.66% and 0% after receiving anti-IL-17 IgG when challenged with the strains PAO1 and PAK, respectively (Figures 10(e) and 10(f)).

## 4. Discussion

Pulmonary infections due to *P. aeruginosa* have emerged as a serious challenge for medical therapy, mainly due to the paucity of new effective antibiotic classes and high morbidity and mortality [35]. This terrible situation has motivated attention in the vaccine, a possible approach to combat *P. aeruginosa*. In addition, the complex pathogenicity of *P. aeruginosa* and the diverse function of its virulence factors represent major obstacles to the development of effective universal vaccines [36]. For full-fledged protection against various *P. aeruginosa* infection, incorporation of innate and adaptive immune system is crucial. In lung tissue, a combination of opsonizing antibodies and inflammatory responses of the innate immune cell is mediated full-fledged protection against *P. aeruginosa* [34]. Thus, antigens in an effective vaccine against *P. aeruginosa* infections should induce both humoral and cellular immune responses. Although several *P. aeruginosa* antigens have so far been evaluated as *P. aeruginosa* vaccine candidates, flagellin proteins hold a greatest promise because these *P. aeruginosa* components evoke *in vivo* immune responses which in turn promote opsonophagocytic activity [25]. They are also vital for *P. aeruginosa* survival during infection and are expressed by the majority of clinical *P. aeruginosa* strains [26]. Moreover, flagellin promotes marked increases in T and B lymphocyte recruitment to secondary lymphoid sites, increasing the likelihood of these cells encountering their specific antigen, and can also directly stimulate CD4^+^ and CD8^+^ T cells [14, 15].

Here, we have shown that bivalent flagellin vaccine provided significant homologous and heterologous protection against fatal *P. aeruginosa* pneumonia and it was associated with antiopsonophagocytic killing and antimotility activities as well as an effector IL-17 cytokine response. Indeed, the antiopsonophagocytic killing and antimotility activities provided by bivalent flagellin antiserum to blunt the pathogenesis and invasive progression of infection coupled with the effector cytokines of Th17 cells augmented a broader spectrum of protective activity against *P. aeruginosa*-induced fatal pneumonia. Evaluation of various aspects of mouse immune response and protective efficacy of the vaccine in a murine acute pneumonia model indicated robust protection against potentially lethal *P. aeruginosa* infections.

The cytokine secretion of splenic T cells indicated that bivalent flagellin could induce efficient Th17 type cytokines by antigen stimulation in combination with the findings that IgG1 and IgG2a subtypes in the serum of immunized mice further confirm that flagellin evokes the development of humoral and cell-mediated immune responses and acquires greater efficacy than monovalent flagellin to provide protection against different flagellated strains of *P. aeruginosa*. These data suggested that collaboration of CD4^+^ T cells with the capacity to produce IL-17 and opsonic antibodies is required for protective immunity against acute *P. aeruginosa* pneumonia [37, 38]. The protective immune response following *P. aeruginosa* immunization of rats is associated with CD4^+^ T cell-dependent immunity [39]. *P. aeruginosa* immunization of mice pulsed DCs protecting mice against pulmonary infection, depending on the presence of CD4^+^ T cells [38].

It must so be mentioned that IL-17 was recently shown to be a critical factor in a vaccine that induced protection to *P. aeruginosa* [10]. IL-17 mainly mediates its immune regulatory function by promoting the production of antimicrobial peptides from lung epithelia or of proinflammatory cytokines and chemokines, which leads to the attraction of neutrophils and macrophages to the lung and the subsequently increased phagocytosis of bacteria and enhanced clearance of infection [9, 10, 40]. Early increased neutrophil number in bivalent flagellin-immunized mouse lung tissue is associated with a rapid reduction of bacterial load in lung tissue as well as in homologous and heterologous protection. Our data suggest that CD4^+^ T cells may be one of the sources of IL-17 during acute pulmonary *P. aeruginosa* infection and could be a key component of full immunity to lung infection by this pathogen [41, 42]. A recent study has shown that *γδ* T cells produced IL-17 in the lungs as early as 2 h after *Bordetella pertussis* infection [43]. The level of IL-17 increased 6 h after infection, demonstrating that acute pulmonary infection with *P. aeruginosa* rapidly induced IL-17 production in the lungs; this IL-17 may be involved in the innate immune response to the infection [44, 45].

Also, T cells from nasally immunized mice with live-attenuated *P. aeruginosa* vaccine show a high IL-17 levels after antigen stimulation and high number of CD4^+^IL-17^+^Th17 cells in the spleen at 6 h after challenge and mediated protective immune responses [34]. Although the focus of studies examining IL-17 production has largely been on CD4^+^*αβ* T cells, *γδ* T cells have also been shown to be a potent source of IL-17 and, in some cases, produce more IL-17 than *αβ* T cells [46–49]. Interestingly, it has been shown that Th17 cells increase after 8 h of infection with *P. aeruginosa*, and the level of IL-23 also increases in the acute pulmonary *P. aeruginosa* infection [44]. Recent studies suggest that IL-23 promotes the Th17 cell development from effector memory CD4^+^ cells. The major cell type in the lung responsible for the clearance of *P. aeruginosa* is the neutrophil [9, 50]. Protection achieved in the killed *P. aeruginosa*-immunized animals was associated with CD4^+^IL-17^+^Th17 and rapid requirement of neutrophil in the lung [51]. The lower bacterial burden in the liver, spleen, and blood showed that the antibodies to bivalent flagellin could systemically disrupt the dissemination of both *P. aeruginosa* stains in the liver and spleen via the blood by inhibition of bacterial motility at the site of infection, which is the principal mechanism of protection in the bloodstream infections following *P. aeruginosa* acute pneumonia model [17, 18, 52]. Although the antibodies against type a or b flagellin completely inhibited the motility of the homologous strain, they also had slight effects on the heterologous strain. The results of motility inhibition and opsonophagocytic activities indicated that the protective effect of antibodies against either type a or b flagellin is strain specific. Passive transfer of bivalent flagellin antisera protected mice against infection with both flagellated strains of *P. aeruginosa*. The achieved results are also consistent with several investigations that employed a burn wound model to show that passive immunization with antibodies raised to type a or b flagellin protects the infected mice with the homologous strain [53, 54]. The protection provided by antibodies raised against flagellin is highly type specific, and the presence of antibodies to both flagellin types is critical. Thus, antibodies against bivalent flagellin showed a great activity against two different *P. aeruginosa* strains and did not interfere with the individual components for improving the opsonophagocytic killing and immobilizing different flagellated strains of *P. aeruginosa*. Hence, immunotherapy with the antibodies to bivalent flagellin is more successful in providing homologous and heterologous protection than therapy with antibodies raised to each monovalent type a or b flagellin. Also, immunization with our novel bivalent flagellin vaccine does not interfere with the individual components in improving survival or induction of protective antibodies. We acknowledge that our protective efficacy data are contrary to Campodonico et al. [23], who exposed that antibodies against type a or b flagellin had a low protective activity against the homologous strain and no activity against the heterologous strain. It seems feasible that the apparent data discrepancy might be explained by our approach using the full-length type a and b flagellins as the target antigen of the study. In addition, Campodonico et al. separately examined the effects of type a and b flagellins but did not assess bivalent flagellin [23]. Our finding that antibodies against type a or b flagellin show high activity against homologous strains and low activity against a heterologous strain is contrary to a recent report [23], in which it was shown that antitype a flagellin antibodies against homologous *P. aeruginosa* strain have low opsonic killing activity and no killing activity. They also reported that type b flagellin antibodies have no opsonic killing activity against either homologous or heterologous strain [23]. The apparent discrepancy may partially be explained by utilizing a mixture of full-length type a and b flagellins (containing N′ and C′-terminal domains) as immunogen, rather than flagellin subunits, and might account for the protective antibody response observed in our study. It is also worth mentioning that several reports have shown that both N′- and C′-termini of flagellin protein have proinflammatory motifs [55, 56] and both trigger activation of the innate immune response via TLR5 that enhances protective inflammatory response and enhance recruitment of PMNs and facilitate *Pseudomonas* clearance postinfection [57]. Also, previous studies have shown that flagellin mutant bacteria elicited severe mucosal damage by a mechanism that also appears to involve the induction of apoptosis in the epithelial cells via inhibition of NF-*κ*B activation [58, 59]. The TLR5 activation via flagellin may prevent bacteria to evade the host's innate immune responses and alter the level of tissue damage associated with late-stage bacterial infection [60]. After colonization, *P. aeruginosa* downregulates the expression levels of flagella to evade from the TLR5-mediated host's innate immune activation [61]*. H. pylori* may have evolved to express inactive flagellin to prevent activation of the TLR5-mediated host's mucosal immunity, enabling persistent infection in the stomach epithelia [62]. Also, Campodonico et al.'s 2011 report demonstrated that conjugation of polymannuronic acid (PMA) to type a flagellin enhanced its immunogenicity via TLR5, indicating desirable protective antibodies were elicited and conserved mechanism of innate immune resistance to *P. aeruginosa* [63]. These observations have important implications for use of flagellin vaccine rather than flagella against flagellated pathogens. It seems that activation of TLR5 may induce the protective immunity against *P. aeruginosa* and decrease the risk to acquire infections from other flagellated bacteria that activate TLR5 [23, 63]. Lastly, we acknowledge that our findings are contrary to the 2010 report [23], which concluded that flagella are a better candidate to provide protection against infection because they observed that antitype a flagellin and antitype b flagellin antibodies have poor protective activity against homologous or heterologous *P. aeruginosa* strains. Several reports, however, have demonstrated that flagellin-induced antibodies raised against a or b type flagellins have protective activity in various murine infection models [53, 64, 65]. Indeed, one novel feature of our approach is targeting both domains of full-length type a and b flagellins, as target antigen, protective anti-flagellin antibodies, as well as inducing CD4^+^IL-17^+^Th17 cell response among immunized mice indicating an active clonal expansion and a neutrophil response that leads to higher survival (protective) rates among immunized mice than control mice.

It is also worth indicating that the active immunization with type a or b flagellin provided significantly protected mice against the heterologous strain than passive therapy with antibodies raised to type a or b flagellin. This phenomenon indicated that CD4^+^IL-17^+^Th17 cell-mediated immune response might be responsible for the anti-infection activity afforded by the bivalent flagellin when antiopsonophagocytic killing and antimotility antibody levels were low. Also, *in vivo* CD4^+^ T cell depletion during immunization diminished the bivalent flagellin or type a or b flagellin vaccine-based protection against *P. aeruginosa* infection. However, the depletion of CD8 lymphocytes showed no abrogation of the anti-infection activity against the *P. aeruginosa* strains. The result of present study demonstrated the fact that both adaptive (CD4^+^ T cells and antibodies) and innate (macrophage and neutrophil) effectors are required for protective immunity against *P. aeruginosa* strains. T cell proliferation and IL-17 secretion response assays were confirmatory data showing the evidence for a heterologous T cell response among immunized flagellin a or b mice indicating an active clonal expansion and a neutrophil response that leads to higher survival (protective) rates among immunized mice than control mice. A recent study demonstrated that protection mediated by PopB immunization against *P. aeruginosa* pneumonia is associated with increased IL-17 cytokine whereas antisera to PopB had neither opsonophagocytic nor anticytotoxic activity [9]. An important feature of a desirable vaccine for *P. aeruginosa* is the ability to induce IL-17 for rapid recruitment of neutrophils to the site of infection, where they can mediate opsonophagocytic bacterial killing. In a recent research [25], we demonstrated that bivalent flagellin vaccine could confer protection against burn wound infection with different flagellated strains of *P. aeruginosa*, by eliciting an effective humoral immune response, including improved opsonophagocytic killing and an immobilization of the pathogen at the wound site. Following burn injury, the failure of local and systemic cellular immune responses allows pathogenic bacteria to disseminate systemically from the site of infection to the bloodstream and easily escalate into sepsis. Thus, it seems that protective immune response against *P. aeruginosa* burn wound infection is mediated by opsonic antibodies. Hence, flagellin is a unique feature because of its ability to induce both cellular and humoral immune responses, which in turn provides protection against different *P. aeruginosa* infections.

Taken together, these results offer evidence that bivalent flagellin vaccine can confer protection against different flagellated strains of *P. aeruginosa* infection in a burn wound model by eliciting effective cellular and humoral immune responses, including induction of IL-17 and improved opsonophagocytic killing. Thus, reducing the systemic pathogen dissemination caused an increase in the survival of mice infected with *P. aeruginosa*. Moreover, these data further confirmed that targeting both types of flagellin with antibodies resulted in significantly improved efficacy in comparison with each monovalent antigen with the potential of broadening the coverage against *P. aeruginosa* strains which might not express one of the two targets. We maintain optimism that an effective *P. aeruginosa* bivalent flagellin vaccine may eventually be developed and tested in humans toward reducing the morbidity and mortality associated with lung infections caused by *P. aeruginosa*.
